# Supplementary material for: PVR and ICAM-1 on Blast Crisis CML Stem and Progenitor Cells with TKI Resistance Confer Susceptibility to NK Cells
Source: Cancers (Basel). 2020 Jul 16;12(7):1923. doi: 10.3390/cancers12071923 (PMC7409178; doi:10.3390/cancers12071923)
Supplement: Supplementary file 1 [file cancers-12-01923-s001.pdf]

Supplementary Materials: PVR and ICAM-1 on Blast Crisis CML Stem and Progenitor Cells with TKI Resistance Confer Susceptibility to NK Cells

Nayoung Kim, Mi-Yeon Kim, Young-Uk Cho, WenYong Chen, Kyoo-Hyung Lee and Hun Sik Kim

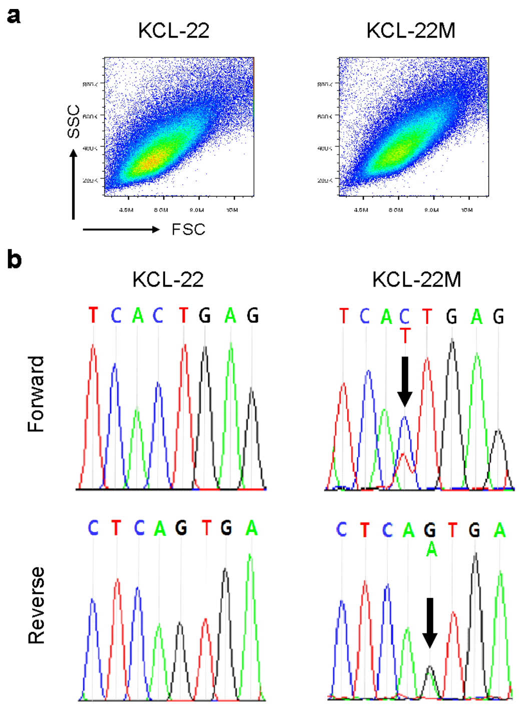

Figure S1. Molecular characterization of BCR-ABL1 T315I mutation in KCL-22M cells. (**a**) Comparison of the size and complexity of KCL-22 and KCL-22M cells. KCL-22M cells exhibited an increase in forward scatter (FSC) and side scatter (SSC) parameters. (**b**) Sequencing analysis of the BCR-ABL1 kinase domain with genomic DNA from KCL-22 and KCL-22M cells. Note the point mutation (arrow) of C to T (forward) and G to A (reverse) that causes the T315I mutation in a mutant allele of KCL-22M cells.

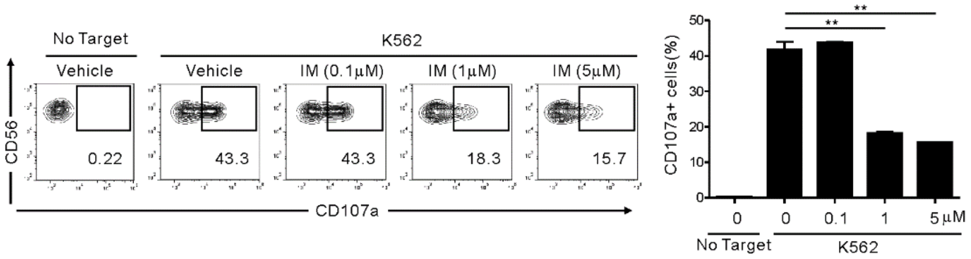

Figure S2. Reduced susceptibility of K562 cells harboring a wild-type BCR-ABL1 to NK cells after IM treatment. K562 cells were pretreated with the indicated doses of IM for 48 h, washed, and then incubated with expanded primary NK cells for 2 h. Shown are representative flow cytometry profile (left) and graph of statistical bar charts (right) demonstrating the percentage of CD107a+ NK cells. The mean values ± s.d. of three independent experiments are shown. ** $p < 0.01$ by Student *t*-test.

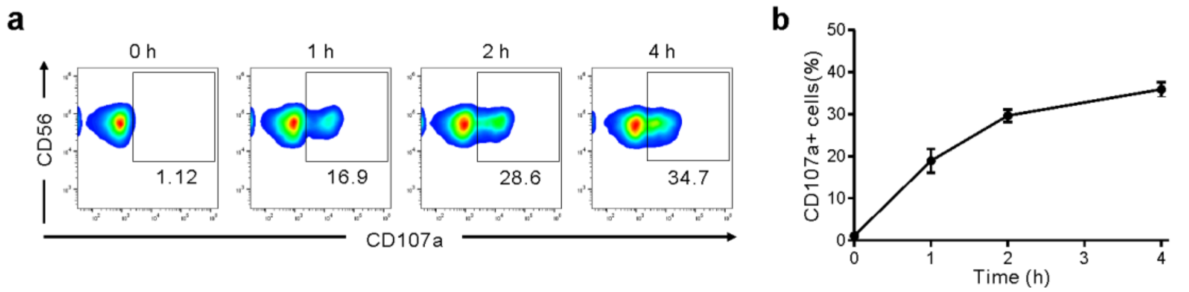

Figure S3. Association of primary CML-BC cell cytolysis with NK cell cytolytic activity. Degranulation assay against primary CML-BC cells with expanded primary NK cells for the indicated times. Shown are representative flow cytometry profile (**a**) and line graph (**b**) showing the cytotoxic degranulation (%) of CD107a+ NK cells. The mean values ± s.d. of three independent experiments are shown.

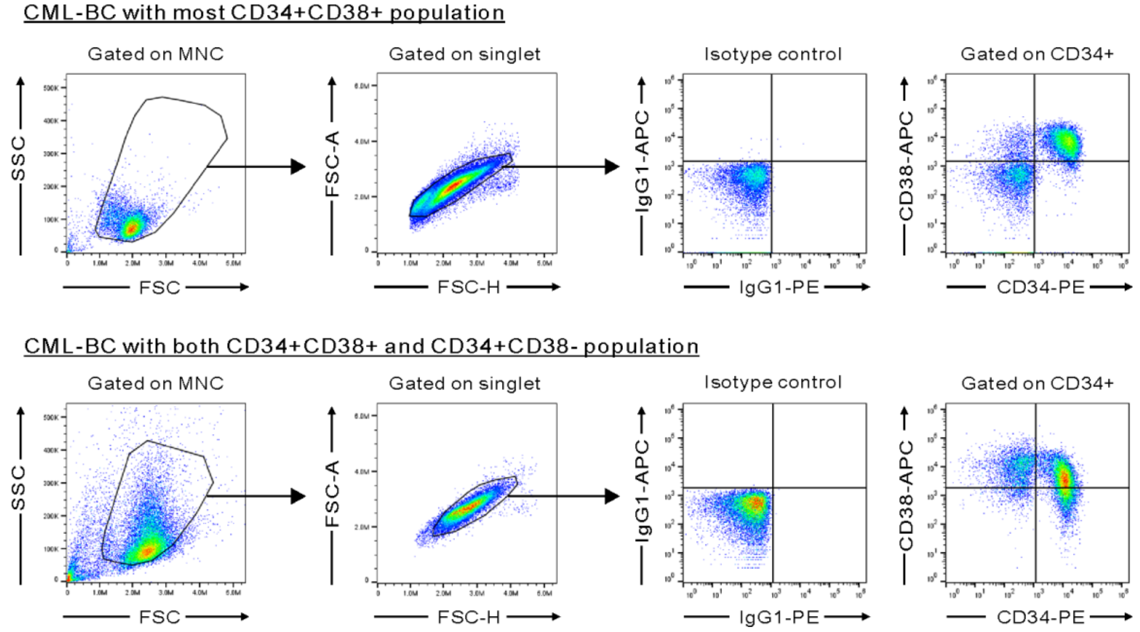

Figure S4. FACS gating strategy. Profiles showing the gating strategy for identifying the $CD34^{+}$ primary CML-BC cells including $CD34^{+}CD38^{-}$ subset within the mononuclear cell (MNC) gate. Gating strategy: forward scatter (FSC) vs. side scatter (SSC) (left panel), then FSC-Height vs. FSC-Area (middle panel), and isotype control or CD34 vs. CD38 (right panel).

Table S1 Characteristics of study patients.

No.	Source	BCR-ABL1 Mutation	Blast Type	TKI-Resistance	CD34/CD38	Karyotype
1	BM	F317L/ E275K	Myeloblast	IM, DAS	Little or no $CD34^+CD38^-$	46,XY,t(9;22)(q34;q11.2)[13]/46,XY[7]
2	PB	T315I	Myeloblast	IM, DAS	Little or no $CD34^+CD38^-$	46,XX,t(9;22)(q34;q11.2)[8]/46,idem.del(9)(p22)[10]/46,XX[2]
3	PB	E255V	Lymphoblast	IM, NIL	$CD34^+CD38^+$/$CD34^+CD38^-$	46,XY,t(9;22)(q34;q11.2)[26]/46,idem,?del(3)(q26.2q27),?del(11)(q23q23)[8]/46,XY[6]
4	PB	E255V	Myeloblast	DAS, NIL	$CD34^+CD38^+$/$CD34^+CD38^-$	46,XY,inv(3)(q21q24),t(9;22)(q34;q11.2)[20]
5	BM	None	Myeloblast	IM	Little or no $CD34^+CD38^-$	55,XX,+4,+8,t(9;22)(q34;q11.2),+10,+del(13)(q12q22),+19,+20,+21,+22,+der(22)t(9;22)[8]/56,idem,+der(22)t(9;22)[2]
6	BM, PB	E255K	Myeloblast	NIL	$CD34^+CD38^+$/$CD34^+CD38^-$	46,XX,t(1;21)(p36.3;q22),t(9;22)(q34;q11.2)[1]/47,sl,-X,+8,+10[25]/48,sdl,+der(22)t(9;22)(q34;q11.2)[4]
7	PB	None	Myeloblast	NIL, DAS, PON	$CD34^+CD38^+$/$CD34^+CD38^-$	Not available

Abbreviations: BM, bone marrow; PB, peripheral blood; TKI, tyrosine kinase inhibitor; IM, imatinib; DAS, dasatinib; NIL, nilotinib; PON, ponatinib.

© 2020 by the authors. Licensee MDPI, Basel, Switzerland. This article is an open access article distributed under the terms and conditions of the Creative Commons Attribution (CC BY) license (http://creativecommons.org/licenses/by/4.0/).
